# The impact of Ramadan on visits related to diabetes emergencies at a tertiary care center

**DOI:** 10.1186/s12873-021-00555-8

**Published:** 2021-12-23

**Authors:** Abdullah M. AlZahrani, Mawaddah M. Zawawi, Naif A. Almutairi, Ammar Y. Alansari, Amina A. Bargawi

**Affiliations:** 1 King Abdullah International Medical Research Center, Department of family medicine, King Saud bin Abdulaziz University for Health Sciences, King Abdulaziz Medical City, Jeddah, Saudi Arabia; 2 Family medicine resident- fourth year, King Abdulaziz Medical City, Jeddah, Saudi Arabia; 3 Emergency medicine resident- fourth year, King Abdulaziz Medical City, Jeddah, Saudi Arabia; 4 King Saud bin Abdulaziz University for Health Sciences, Jeddah, Saudi Arabia

**Keywords:** Diabetes, Ramadan, Emergency visit, Fasting

## Abstract

**Background:**

Ramadan is the ninth month of the Islamic calendar were Muslims fast from dawn until sunset. This prolonged fasting period might have an impact on patients with diabetes and their disease control. This study aimed to determine the variation in visits at the Emergency Room department (ER) during Ramadan in comparison with other lunar months at a tertiary care hospital in Jeddah city in relation to the diabetes emergencies.

**Methodology:**

A retrospective cross-sectional study was conducted using electronic medical record review of patients with diabetes emergencies who visited ER of a Military hospital, from 9th to 11th lunar months during 2017–2018. Diabetes patients who visited ER and aged more than 18 years old were included. Frequency of ER visits, sociodemographic characteristics and clinical features were determined. Chi-square test, Student ‘s t-test and one-way ANOVA at *p* < 0.05 were used in assessing associations between variables.

**Results:**

Within the selected study period, a total of 24,498 admissions were recorded in ER. The prevalence of diabetes emergencies visits was only 0.84%. Based on inclusion criteria, a total of 133 subjects were included in the study (54.1% men, 45.9% women). Majority of whom (73.7%) were on insulin therapy, and more than half of whom (51.9%) were type 2 diabetes. There was a significant difference (*p* = 0.001) in the prevalence of diabetes emergencies visits between the three lunar months Shaban, Ramadan and Shawal, 7, 5 and 4%, respectively. However, the highest prevalence was not in Ramadan. Despite some correlations were identified, the study found no significant differences between frequency of ER visits and various demographic, clinical factors and diabetes profile between Ramadan and other both preceding and succeeding lunar month.

**Conclusion:**

In contrary with previous studies, a downward trend of prevalence, from Shaban to Ramadan, to Shawal was found. This indicated that fasting during month of Ramadan does not impact negatively on the diabetes emergencies in comparison with other months. Hyperglycemia among type 2 diabetes and insulin treated patients were recorded the highest feature of diabetes emergences visits during the three months studied with no significant differences between the months. These findings highlight the need of type 2 and insulin treated patients to be thoroughly assess by the Primary Care physicians and in-depth health education and guidance should be given to them.

## Introduction

Ramadan is an important fasting period for Muslims, which lasts for 29 to 30 days and ranges from 14 to 18 h per day in the 9th lunar month. It is mandatory among healthy Muslims past puberty age, as it is deemed as one of the five pillars of Islam [1]. Immense changes of daily habits are observed among Muslims during Ramadan, influencing the physical activity, sleeping cycle, hours allotted for duty, and the amount and kind of meals being eaten [2]. However, this fasting practice can significantly affect diabetic patients, with heightened risk of hypoglycemia, hyperglycemia, diabetic ketoacidosis (DKA), and increased hospital visits due to complications brought forth by diabetes [3]. This change in daily activities during Islam impacts emergency visits in hospitals as brought by varying number of patients between night and day during regional festivals and cultural events, such as Ramadan [2]. In the research conducted by Elbarsha and colleagues, there is a lower number of admissions among patients with diabetes during Ramadan (186) as compared to Dhu al-Qidah (216), which is the 11th lunar month [3]. Likewise, cases of severe hypoglycemia had amplified during Ramadan due to insufficient monitoring and restricted regimen usage, as provided by an epidemiological study conducted across 13 countries [4]. Another study revealed a substantial increment in cases of hypoglycemia during Ramadan as compared to other lunar months, particularly for insulin treated diabetics in comparison to those who use oral hypoglycemic agents (OHA). This is different from the heightened hospitalization rate and incidence caused by DKA in Ramadan with prolonged acidosis as obtained by Abdelgadir and colleagues [5]. This prospective study was conducted by major hospitals in United Arab Emirates, Sudan, Tunisia, and Morocco, involving 167 patients from lunar month before Ramadan (Shaban) until a month after fasting period (Shawal). Type 1 diabetes patients comprised the people group admitted during quarantine, with less than 30% of them receiving diabetes management program in Ramadan. Another study revealed an increment in hyperglycemic episodes evidenced by broad-changing range of blood glucose, in comparison with hypoglycemic episodes [6]. Therefore, there is an augmented necessity for regimen modifications as well as proper lifestyle adjustments among diabetic patients that are deemed appropriate during Ramadan, as unmanaged cases of diabetes can potentially need to serious complications.

Few studies have been conducted to assess the frequency of diabetic cases related to fasting period during Ramadan in Saudi Arabia. As such, this study is aimed at determining variation in visits at the emergency room department (ED) during Ramadan in a tertiary care hospital located in Jeddah. In addition, the occurrence of diabetes emergencies during Ramadan as well as the final outcomes were determined. Likewise, data on diabetes ER visits both preceding and succeeding lunar months were determined for comparison.

## Materials and methods

This cross-sectional study was conducted by reviewing of the medical records and the health information system ‘BESTCare’ between 20th of April 2017 and 31st of December 2018. The basic population consisted of patients aged between 18 and 70 years old with diabetes emergencies who visited ER of King Abdulaziz Medical City, Ministry of National Guards – Health Affairs, Jeddah, Saudi Arabia during the study period. Patients with gestational diabetes going to labor, below 18 years or above 70 were excluded from the study.

Medical record review was conducted for all ER visit for patients with diabetes emergencies under the coding system of International Classification of Diseases version 10 (ICD-10). Monthly and daily ER visit for diabetes patients were established as dependent variables in this study. The following characteristics were determined: (1) sociodemographic factors, such as sex, age, and presence of other diseases, such as ischemia and intra-abdominal sepsis; and (2) clinical features, such as type of diabetes, arrival time, precipitating factors, anti-hyperglycemic treatment type, mean serum sodium, mean venous pH, mean plasma glucose, mean serum urea, as well as final diagnosis.

**Fig. 1:**
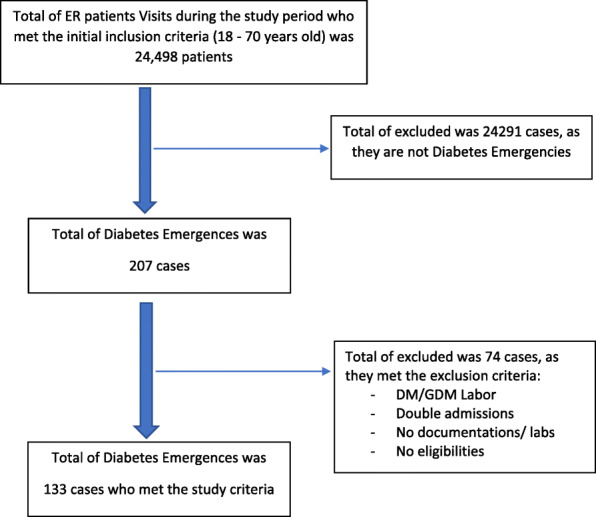
flow diagram for inclusion and exclusion criteria

The total number of ER visits in the selected 3 months during the study period (two years) was high, 24,498 patients, however, the number of identified diabetes emergencies was very low, only 207 cases (0.84%)**.** This limited figure could be justified by the health status of the military hospital’s population, which are mainly fit male military personnel in addition to little proportion of their dependents (parents, grandparents, wives and children). Out of those 207 cases, only 133 cases were included in the study based on the study’s inclusion criteria, this selection process was illustrated by flow diagram for inclusion and exclusion criteria, Fig. 1. Continuous variables were summarized as mean and standard deviation while categorical variables were presented as frequencies and percentages. Categorical variables were correlated using Chi-square test, whereas one-way ANOVA and student’s t-test were used in assessing relationships between dependent variables with the selected continuous independent variables. Data management and analysis was performed using Statistical Package for Social Sciences (SPSS) ver. 24 (IBM Corp., Armonk, NY), at statistical significance of *p* < 0.05.

## Results

Sociodemographic factors were studied for their relationship with ER visit among patients with diabetes during Ramadan and other two lunar months (Shaban and Shawal), as shown in Table 1. Despite that Chi-square test revealed no significant different among various age groups, gender, and comorbidity, however, some differences were observed. During Shaban (lunar month before Ramadan), about 40% of ER patients were aged between 40 and 59 years old and majority of which were female (52.5%). Younger patients aged 18 to 39 years old as well as male patients were reported to visit ER during Ramadan as compared to their counterparts. On the 10th lunar month (Shawal), patients at least 60 years old, mostly males, were reported to visit ER. In these 3 lunar months, majority of them reported presence of other diseases aside from diabetes. The prevalence of diabetes emergencies visits was 0.84%, out of 24,498 ER visits, only 207 cases were diabetes emergencies. The ER visits that related to diabetes emergencies for the 3 lunar months was found to have a downward trend from Shaban to Ramadan to Shawal, (7, 5 and 4% respectively), as provided in Table 2. However, overall, diabetes emergencies that met the study inclusion criteria was 0.5% of the ER visits for 3 lunar months. Statistical analysis showed substantial variation in the number of ER visits among diabetes patients during these 3 lunar months as shown in Table 2. This variation is mainly accounted by significantly higher number of type 2 diabetes patients admitted one month before Ramadan. During Ramadan, there is no significant difference in the number of both type 1 and type 2 admitted patients. A month after Ramadan, type 1 diabetes patients were admitted more than type 2. Table 3 shows that more than half of the diabetes emergences patients were type 2 (52.3%), almost three quarter of them were on insulin therapy (74.8%) and majority of their clinical presentation was hyperglycemia (72.2%). Infections was predominant contributing factor for ED visits with uncontrolled diabetes which was noticed in Shawal (62.5%) and Ramadan (33.3%). On the other hand, few ED cases were represented as nonadherence in Ramadan, 10% missed insulin doses and 5% had missed a meal. Furthermore, over than 20% of patients have reported no obvious cause or precipitated factor that contribute to ED visits in the whole 3 months. Diabetes mellites is associated with increased risk of cardiovascular disease; Ischemia and infarctions are the common cases in ED visits, in both Shaban and Ramadan more than 20% of uncontrolled diabetes had ischemia. For diabetes patients who visited ER during Shaban and Ramadan, there is a significant difference in terms of final ER decision and diabetes type. For final ER decision, half of diabetes patients were admitted to hospital during Ramadan, as compared to about 72.9% of the diabetes patients admitted during Shaban. In terms of diabetes type, almost half (47.5%) of diabetic patients during Ramadan were found to have type2 diabetes, while 70% of diabetic patients during Shaban had type 1 diabetes. Other variables, such as precipitating factors, type of antidiabetic medication, clinical presentation, and arrival time at ER during Shaban and Ramadan were found to have no statistical variation, as shown in Table 4. On the other hand, arrival time at ER was found to be substantially different when comparing Ramadan and Shawal months. Month, as shown in Table 5. About 40.7% patients with diabetes visited ER at day shift during Ramadan whereas 61.8% of them visited ER at night shift during Shawal. Type 2 diabetes patients frequently visited ER during Ramadan while type 1 diabetes patients visited during Shawal. Other variables from these 2 lunar months were found to have no statistical difference. Comparison of continuous variables, such as length of hospital stay, plasma glucose, venous pH, serum sodium, potassium, and urea, during these 3 lunar months were also evaluated as provided in Tables 6, 7 and 8. One-way ANOVA revealed no statistical variation between these variables during these lunar months. Moreover, Student’s t-test for clinical characteristics revealed no statistical difference between these variables during Ramadan as well as both preceding and succeeding lunar month.

**Table 1:** Socio-demographic characteristics of diabetes patients who visited ER in a tertiary care hospital in Jeddah, Saudi Arabia from 2016 to 2018. (*n* = 133)

		SHAABAN		RAMADAN		SHAWAL		Chi-square	***p*** value
		n	%	n	%	n	%		
**Age group**	18–39	17	28.8%	17	42.5%	9	26.5%	5.789	0.215
	40–59	24	40.7%	8	20.0%	11	32.4%		
	60 +	18	30.5%	15	37.5%	14	41.1%		
Total		59	100.0%	40	100.0%	34	100.0%		
	Mean ± SD				48.53 ± 17.26				
	Minimum				18				
	Maximum				72				
	Median				54				
	Interquartile range				31				
**Gender**	Male	28	47.5%	25	62.5%	19	55.9%	2.229	0.328
	Female	31	52.5%	15	37.5%	15	44.1%		
Total		59	100.0%	40	100.0%	34	100.0%		
**Other Diseases**	Yes	38	64.4%	24	60.0%	23	67.6%	0.477	0.788
	No	21	35.6%	16	40.0%	11	32.4%		
Total		59	100.0%	40	100.0%	34	100.0%		

*Significant at *P*-value less than 0.05

**Table 2:** Prevalence of diabetic emergencies in three lunar months from 2016 to 2018 (*n* = 24,498)

	Total of ER visits	Diabetic Emergencies	% of ER visits by patients with diabetes	% of ER visits among diabetic patients during 3 lunar months	*P* value
SHAABAN	8547	59	0.7%	44.4	0.001*
**RAMADAN**	**8177**	**40**	**0.5%**	30.1	
SHAWAL	7774	34	0.4%	25.6	
**Total**	**24,498**	**133**	**0.5%**	100	

*Significant at *P*-value less than 0.05

**Table 3:** Clinical characteristics of diabetic with ER visits during the three lunar months (*n* = 133)

		SHAABAN		RAMADAN		SHAWAL		Total		Chi-square	***p*** value
		n	%	n	%	n	%	N	%		
Type of Antidiabetic medications	OHA	7	12.1%	1	2.5%	0	0.0%	8	6.1	12.9	0.045*
	Insulin	39	67.2%	31	77.5%	28	84.8%	98	74.8		
	Mixed	11	19.0%	7	17.5%	2	6.1%	20	15.3		
	None	1	1.7%	1	2.5%	3	9.1%	5	3.8		
Total		58	100.0%	40	100.0%	33	100.0%	131	100		
Type of Diabetes	Type I	15	25.4%	16	40.0%	15	45.5%	46	34.8	31.48	0.001*
	Type II	42	71.2%	19	47.5%	8	24.2%	69	52.3		
	GDM	2	3.4%	1	2.5%	0	0.0%	3	2.3		
	Not Known	0	0.0%	4	10.0%	10	30.3%	14	10.6		
Total		59	100.0%	40	100.0%	33	100.0%	132	100		
Arrival Time at ER	Day shift	24	40.7%	16	40.0%	9	26.5%	49	36.8	23.33	0.001*
	Evening shift	26	44.1%	12	30.0%	4	11.8%	42	31.6		
	Night shift	9	15.3%	12	30.0%	21	61.8%	42	31.6		
Total		59	100.1%	40	100.0%	34	100.1%	133	100		
Precipitating factors	Missed Dose	12	20.3%	4	10.3%	2	6.3%	18	13.8	20.51	0.025*
	Infection	15	25.4%	13	33.3%	20	62.5%	48	36.9		
	No Obvious cause	16	27.1%	9	23.1%	7	21.9%	32	24.6		
	First time	3	5.1%	1	2.6%	1	3.1%	5	3.8		
	Missed Meal	0	0.0%	2	5.1%	1	3.1%	3	2.3		
	Ischemia/infarction	13	22.0%	10	25.6%	1	3.1%	24	18.5		
	Intra-Abdominal Sepsis	0	0.0%	0	0.0%	0	0.0%	0	0.0		
Total		59	99.9%	39	100.0%	32	100.0%	130	100		
Clinical presentation	Hypoglycemia	1	1.7%	1	2.5%	2	5.9%	4	3.0	1.355	0.852
	Hyperglycemia	43	72.9%	29	72.5%	24	70.6%	96	72.2		
	DKA	15	25.4%	10	25.0%	8	23.5%	33	24.8		
Total		59	100.0%	40	100.0%	34	100.0%	133	100		
Final Diagnosis in the ER	Admitted to hospital	43	72.9%	20	50.0%	18	52.9%	81	60.9	11.822	0.159
	Refer to another facility	0	0.0%	2	5.0%	1	2.9%	3	2.3		
	Rejection of treatment	1	1.7%	0	0.0%	1	2.9%	2	1.5		
	Death	0	0.0%	0	0.0%	0	0.0%	0	0.0		
	Discharge from ER	13	22.0%	18	45.0%	13	38.2%	44	33.1		
	Admitted to ICU	2	3.4%	0	0.0%	1	2.9%	3	2.3		
	Admitted to CCU	0	0.0%	0	0.0%	0	0.0%	0	0.0		
Total		59	100.0%	40	100.0%	34	99.8%	133	100		

Pearson Chi-square test, *significant at *P*-value less than 0.05

**Table 4:** Clinical characteristics of diabetic with ER visits during Shaaban and Ramadan (*n* = 99)

		RAMADAN		SHAABAN		Chi-square	***p*** value
		n	%	n	%		
Type of Antidiabetic medications	OHA	1	2.5%	7	12.1%	3.102	0.376
	Insulin	31	77.5%	39	67.2%		
	Mixed	7	17.5%	11	19.0%		
	None	1	2.5%	1	1.7%		
Total		40	100%	59	100%		
Type of Diabetes	Type I	16	40.0%	15	25.4%	9.750	0.021*
	Type II	19	47.5%	42	71.2%		
	GDM	1	2.5%	2	3.4%		
	Not Known	4	10.0%	0	0.0%		
Total		40	100%	59	100%		
Arrival Time at ER	Day shift	16	40.0%	24	40.7%	3.675	0.159
	Evening shift	12	30.0%	26	44.1%		
	Night shift	12	30.0%	9	15.3%		
Total		40	100%	59	100%		
Precipitating factors	Missed Dose	4	10.3%	12	20.4%	5.648	0.342
	Infection	13	33.3%	15	25.4%		
	No Obvious cause	9	23.1%	16	27.1%		
	First time	1	2.6%	3	5.1%		
	Missed Meal	2	5.1%	0	0.0%		
	Ischemia/infection	10	25.6%	13	22.0%		
	Intra-Abdominal Sepsis	0	0.0%	0	0.0%		
Total		39	100%	59	100%		
Clinical presentation	Hypoglycemia	1	2.5%	1	1.7%	0.079	0.961
	Hyperglycemia	29	72.5%	43	72.9%		
	DKA	10	25.0%	15	25.4%		
Total		40	100%	59	100%		
Final Diagnosis in the ER	Admitted to hospital	20	50.0%	43	72.9%	10.961	0.027*
	Refer to another facility	2	5.0%	0	0.0%		
	Rejection of treatment	0	0.0%	1	1.7%		
	Death	0	0.0%	0	0.0%		
	Discharge from ER	18	45.0%	13	22.0%		
	Admitted to ICU	0	0.0%	2	3.4%		
	Admitted to CCU	0	0.0%	0	0.0%		
Total		40	100%	59	100%		

Pearson Chi-square test, *significant at *P*-value less than 0.05

**Table 5:** Clinical characteristics of diabetic with ER visits during Ramadan and Shawal (*n* = 74)

		RAMADAN		SHAWAL		Chi-square	***p*** value
		n	%	n	%		
Type of Antidiabetic medications	OHA	1	2.5%	0	0.0%	4.299	0.231
	Insulin	31	77.5%	28	84.8%		
	Mixed	7	17.5%	2	6.1%		
	None	1	2.5%	3	9.1%		
Total		40	100%	33	100%		
Type of Diabetes	Type I	16	40.0%	15	45.5%	7.483	0.058*
	Type II	19	47.5%	8	24.2%		
	GDM	1	2.5%	0	0.0%		
	Not Known	4	10.0%	10	30.3%		
Total		40	100%	33	100%		
Arrival Time at ER	Day shift	16	40.0%	9	26.5%	7.981	0.018*
	Evening shift	12	30.0%	4	11.8%		
	Night shift	12	30.0%	21	61.8%		
Total		40	100%	34	100%		
Precipitating factors	Missed Dose	4	10.3%	2	6.3%	9.501	0.091
	Infection	13	33.3%	20	62.5%		
	No Obvious cause	9	23.1%	7	21.9%		
	First time	1	2.6%	1	3.1%		
	Missed Meal	2	5.1%	1	3.1%		
	Ischemia/infection	10	25.6%	1	3.1%		
	Intra-Abdominal Sepsis	0	0.0%	0	0.0%		
Total		39	100%	32	100%		
Clinical presentation	Hypoglycemia	1	2.5%	2	5.9%	0.544	0.762
	Hyperglycemia	29	72.5%	24	70.6%		
	DKA	10	25.0%	8	23.5%		
Total		40	100%	34	100%		
Final Diagnosis in the ER	Admitted to hospital	20	50.0%	18	52.9%	2.777	0.596
	Refer to another facility	2	5.0%	1	2.9%		
	Rejection of treatment	0	0.0%	1	2.9%		
	Death	0	0.0%	0	0.0%		
	Discharge from ER	18	45.0%	13	38.4%		
	Admitted to ICU	0	0.0%	1	2.9%		
	Admitted to CCU	0	0.0%	0	0.0%		
Total		40	100%	34	100%		

Pearson Chi-square test, *significant at *P*-value less than 0.05

**Table 6:** Variation in diabetic profile during ER visits in three lunar months (*n* = 133)

	SHAABAN	RAMADAN	SHAWAL	F	*p value*
	Mean ± SD	Mean ± SD	Mean ± SD		
Length of stay in hospital	4.16 ± 7.94	2.73 ± 5.97	10.59 ± 37.03	1.658	0.195
Plasma glucose	20.93 ± 10.34	22.97 ± 10.85	20.18 ± 8.03	0.812	0.446
Sodium	133.05 ± 4.87	133.05 4.87	132.18 ± 4.19	0.332	0.718
Potassium	4.50 ± 0.70	4.77 ± 0.63	4.56 ± 0.53	2.257	0.109
Urea	8.83 ± 7.12	7.90 ± 4.30	8.63 ± 5.28	0.309	0.735
Venous PH	7.29 ± 0.12	7.20 ± 0.46	7.23 ± 0.24	0.599	0.552

F test value for ANOVA test, * significant at *P*-value less than 0.05

**Table 7:** Variation in diabetic profile during ER visits in Shaaban and Ramadan (*n* = 99)

	RAMADAN	SHAABNAN	t	*p value*
	Mean ± SD	Mean ± SD		
length of stay in hospital	2.73 ± 5.97	4.16 ± 7.94	−0.967	0.336
plasma glucose	22.97 ± 10.85	20.93 ± 10.34	0.943	0.348
sodium	133.05 4.87	133.05 ± 4.87	0.347	0.729
potassium	4.77 ± 0.63	4.50 ± 0.70	1.991	0.049
urea	7.90 ± 4.30	8.83 ± 7.12	−0.746	0.457
venous PH	7.20 ± 0.46	7.29 ± 0.12	−0.979	0.332

t test value for independent sample t test, * significant at *P*-value less than 0.05

**Table 8:** Variation in diabetic profile during ER visits in Ramadan and Shawal (*n* = 74)

	RAMADAN	SHAWAL	t	*p value*
	Mean ± SD	Mean ± SD		
length of stay in hospital	2.73 ± 5.97	10.59 ± 37.03	−1.325	0.189
plasma glucose	22.97 ± 10.85	20.18 ± 8.03	1.236	0.220
sodium	133.05 4.87	132.18 ± 4.19	0.820	0.415
potassium	4.77 ± 0.63	4.56 ± 0.53	1.534	0.129
urea	7.90 ± 4.30	8.63 ± 5.28	−0.656	0.514
venous PH	7.20 ± 0.46	7.23 ± 0.24	−0.292	0.771

t test value for independent sample t test, * significant at *P*-value less than 0.05

## Discussion

In this study, there is no significant correlation between frequency of ER visits and various demographic and clinical factors. A comparative study on the effect of Ramadan on ER visits during and 30 days after the said lunar month revealed significantly higher number of patients admitted during Ramadan, in comparison to the succeeding lunar month. In terms of demographic and clinical features, there is no statistical significance between the two periods. Likewise, there is no significant variation in terms of clinical characteristics of patients as well as the admission frequency for injuries, neurological and respiratory diseases during Ramadan [7]. Categorical variables of various diabetic ER patients were assessed in this study, as shown in Tables 3, 4 and 5. Chi-square test of these variables showed substantial variation among several variables such as type of antidiabetic medications, diabetes type, arrival time at ER, and precipitating factors. However, clinical presentation and final decision in the ER do not statistically vary during these lunar months (Table 3). Despite the lowering number of admissions from before until after Ramadan, more insulin-dependent patients were admitted as Ramadan passes, 77.5% of patients were admitted in Ramadan whereas 82% were in Shawal. The fact that patients were on insulin reflect that they are either type 1 or uncontrolled type 2 diabetes mellitus. Most patients in the study were hyperglycemic and majority were insulin dependent (Table 3). In addition, more patients (around 40%) were admitted at daytime during Ramadan as fasting occurs. This means that patients could not withstand fasting during the day. Infections was predominant contributing factor for ER visits with uncontrolled diabetes which was noticed in Shawal (62.5%) and Ramadan (33.3%). On the other hand, few ER cases were represented as nonadherence in Ramadan, 10% missed insulin doses and 5% had missed a meal. Furthermore, over than 20% of patients have reported no obvious cause or precipitated factor that contribute to ER visits in the whole 3 months. A related study on analyzing ER patients flow in a tertiary hospital in Abu Dhabi, United Arab Emirates (UAE) during Ramadan in comparison to non-Ramadan days from 2014 to 2016 [ 8]. With 45,116 ER patient visits, increased visits were recorded during non-Ramadan days. During Ramadan, more than half of the patients (53%) were present during fasting period, which is significantly different from that of non-fasting period (47%). Another study evaluated the arrival patterns as well as characteristics of patients admitted in a pediatric and adult ER in UAE during Ramadan from 2010 to 2013. There is statistical significance for the admission pattern during both Ramadan and non-Ramadan days [9]. ER visits are intrinsically variable and unpredictable. As such, better allocation of resources can be established by predicting ER usage. However, there is limited data on variation in local ER visit pattern during Ramadan. For a 4-year period, majority of the ER visits (57.14%) happened during day shift. However, about 3 in every 5 patients visited ER at night shift during Ramadan. As such, proper allocation of resources during Ramadan is necessary among Muslim countries for efficient management of surge of ER patients at night shift [10]. However, results of this study showed higher number of visits among diabetics during day shift, mostly those with type 2 diabetes. Elbarsha and colleagues determined the impact of fasting on diabetic patients admitted at hospitals and their final outcomes during Ramadan in comparison to the 11th lunar month, which is a non-fasting period. With about 60% of admitted patients fasting during Ramadan, significantly higher incidence of acute coronary syndrome was obtained for fasting patients. However, non-fasting patients had statistically significant in-hospital mortality, as they had more complications that made them ineligible for fasting [3]. Decline in ER visits were also observed from Shaban until Shawal. In Beirut, Lebanon, a study on ER visits, emergency incidences clinical outcomes during Ramadan was determined in a tertiary care center. Mean daily ER admission during non-Ramadan months was higher as compared to Ramadan period, whereas longer hospital stay period was recorded during Ramadan. Although most data are comparable, lower admission rates were observed for patients with stroke or acute coronary syndrome during Ramadan. Furthermore, both ER bounce-back rates and death at ER discharge were amplified during Ramadan. During Ramadan, the following conditions might be experienced by ERs: longer stay in period, decline in admissions, as well as possible worse outcomes. Fluctuations in ER visits connected with common conditions are not anticipated [3]. The results of this research project a different trend as shorter length of stay in hospital were recorded during Ramadan (Table 6). For insulin-dependent fasting diabetics, Gad and others revealed no substantial variation in terms of glycemic control (HbA1c) between multiple daily injections (MDI) and continuous subcutaneous insulin infusion (CSII). However, incidence of DKA and hypoglycemia as well as glucose profile were not assessed due to insufficient data [11]. Similar trend was observed as no significant difference was observed in plasma glucose levels from Shaaban until Shawal. A different study revealed glucose level increments during Ramadan compared to pre-Ramadan level for Type 2 diabetes patients followed by significant decline in terms of glucose level after Ramadan. For triglyceride and cholesterol levels, a similar trend was observed although the difference was not statistically substantial. This study revealed that glucose, triglyceride, and cholesterol levels return to pre-fasting levels after Ramadan [12]. Similarly, results showed an increase in glucose levels during Ramadan, with a corresponding return to pre-fasting levels in Shawal. Hassanein and others evaluated the fasting participants, fasting period, hypoglycemic event rate, glycemic control, and lifestyle patterns among type 2diabetes Middle Eastern and North African participants during Ramadan 2016. The average fasting period lasted for 27.7 ± 5.0 days, with almost 60% of the participants fasted during the whole Ramadan period. Substantial development in FPG, HbA1c, and PPG was observed after Ramadan. Confirmed hypoglycemia amplified substantially until Ramadan period, which was found to depend on treatment regimen used. Incidence of severe hypoglycemia also increased significantly up until Ramadan period. Lifestyle changes were reported by majority of the participants during Ramadan [13].Fasting during Ramadan period can also impact medication adherence among diabetes patients. A certain research evaluated the patient-guided modifications, either twice or once daily, of OAC medication and its possible consequences, as compared to scheduled regular intake. More than half of the participants (53.1%) changed their intake schedule during Ramadan. Likewise, majority of the patients took the medication twice daily. Around 10% of diabetic patients were admitted during Ramadan, which made patient-guided modification a significant variable for hospital admission. During Ramadan, OAC intake with patient-guided amendment is generally observed and accompanied with heightened risk of admission in hospitals. Patient education and effective OAC intake arrangement are deemed advisable during Ramadan [14]. Alkandari and colleagues stated that most of health-specific results associated with Ramadan fasting are mixed. These variations could be indicated by the various factors, such as number of smoking patients, daily fasting period, food, and lifestyle variations, as well as intake of oral medications and intravenous fluids. Meals taken during nighttime before dawn, together with liver glycogen storage, maintains glucose homeostasis during Ramadan. On the other hand, physical activity, body weight changes, as well as the quantity and quality of food intake determines the variation in serum lipids. According to them, Ramadan fasting may be observed by type 2 diabetes patients who are well-controlled and compliant. Healthy individuals can observe Ramadan fasting, but consultation with physicians is required for those with diseases [15]. Lipid profile, glycemic control, dietary intake and weight can significantly be affected by deviations in sleeping patterns, daily physical activities, as well as dietary habits during Ramadan. Hui and Devendra have suggested pre-Ramadan assessment among patients to evaluate the risks associated with fasting. In addition, timing and dosage adjustments with respect to insulin intake, as well as several hypoglycemic agents, it might be necessary during Ramadan with consultation form medical experts [16].

### Limitations

This study was conducted in only one setting, a tertiary military hospital (National Guard Hospital) in Jeddah. Therefore, patients’ access was restricted to those who have the eligibility in their health care. Accordingly, the sample size of the study was very small (*n* = 133), when considering the high prevalence of diabetes mellitus and the high number of patients with diabetes that visited the ER in different hospitals in Saudi Arabia.

## Conclusion

In contrary with previous studies, a downward trend of prevalence, from Shaban, to Ramadan, to Shawal was found. This indicated that fasting during month of Ramadan does not impact negatively on the diabetes emergencies in comparison with other months. Hyperglycemia among Type 2 diabetes and insulin treated patients were recorded the highest feature of diabetes emergences visits during the three months studied with no significant differences between the months. These findings highlight the need of type 2 and type 1 diabetes patients to be thoroughly assess by the Primary Care physicians and in-depth health education and guidance should be given to them.

## Data Availability

Available upon request.

## Abbreviations

DKA: Diabetic ketoacidosis
OHA: Oral hypoglycemic agents
ED: Emergency Room department
ER: Emergency room
SPSS: Statistical Package for Social Sciences
MDI: Multiple daily injections
CSII: Continuous subcutaneous insulin infusion
OAC: Oral anticoagulant
